# Reduction in the Incidence Density of Pressure Injuries in Intensive Care Units after Advance Preventive Protocols

**DOI:** 10.3390/healthcare11152116

**Published:** 2023-07-25

**Authors:** Ru-Yu Lien, Chien-Ying Wang, Shih-Hsin Hung, Shu-Fen Lu, Wen-Ju Yang, Shu-I Chin, Dung-Hung Chiang, Hui-Chen Lin, Chun-Gu Cheng, Chun-An Cheng

**Affiliations:** 1 Department of Nursing, Taipei Veterans General Hospital, Taipei 112201, Taiwan; rylien@vghtpe.gov.tw (R.-Y.L.); hungsh@vghtpe.gov.tw (S.-H.H.); sflu@vghtpe.gov.tw (S.-F.L.); wjyang2@vghtpe.gov.tw (W.-J.Y.); sichin@vghtpe.gov.tw (S.-I.C.); 2 School of Nursing, National Yang Ming Chiao Tung University, Taipei 112304, Taiwan; 3 Department of Exercise and Health Sciences, University of Taipei, Taipei 111036, Taiwan; wangcy@vghtpe.gov.tw; 4 School of Medicine, National Yang Ming Chiao Tung University, Taipei 112304, Taiwan; dhchiang2@vghtpe.gov.tw; 5 Department of Critical Care Medicine, Taipei Veterans General Hospital, Taipei 112201, Taiwan; 6 Division of Trauma, Department of Emergency Medicine, Taipei Veterans General Hospital, Taipei 112201, Taiwan; 7 Department of Nursing, Chang Jung Christian University, Tainan 711301, Taiwan; 8 School of Nursing, College of Nursing, Taipei Medical University, Taipei 11031, Taiwan; cecilia@tmu.edu.tw; 9 Department of Emergency Medicine, Taoyuan Armed Forces General Hospital, Taoyuan 32549, Taiwan; 10 Department of Emergency Medicine, Tri-Service General Hospital, National Defense Medical Center, Taipei 11490, Taiwan; 11 Department of Emergency, Wan Fang Hospital, Taipei Medical University, Taipei 11696, Taiwan; 12 Department of Neurology, Tri-Service General Hospital, National Defense Medical Center, Taipei 11490, Taiwan

**Keywords:** pressure injury, bundle care, incidence density reduction

## Abstract

(1) Background: Patients who are critically ill or undergo major surgery are admitted to intensive care units (ICUs). Prolonged immobilization is the most likely cause of pressure injuries (PrIs) in the ICU. Previous studies of Western populations found that effective protocols could reduce the incidence of PrIs, and the efficacy of systemic targeted intervention protocols in preventing PrIs in the Chinese population needs to be surveyed. (2) Methods: We reviewed cases of PrIs in the ICUs of Taipei Veterans General Hospital from 2014 to 2019. The ICU nurses at the hospital began to implement targeted interventions in January 2017. The incidence density of PrIs was calculated by dividing the number of PrIs by person days of hospitalizations in the pre-bundle (2014–2016) and post-bundle (2017–2019) stages. Poisson regression was performed to compare the trend of incidence densities. (3) Results: The incidence density of PrIs was 9.37/1000 person days during the pre-bundle stage and 1.85/1000 person days during the post-bundle stage (*p* < 0.001). The relative risk (RR) was 0.197 (95% confidence interval: 0.149–0.26). The incidence densities of iatrogenic PrIs and non-iatrogenic PrIs decreased as the RRs decreased. (4) Conclusions: Targeted interventions could significantly reduce the incidence of PrIs. Healthcare providers must follow the bundle care protocol for PrI prevention to improve the quality of healthcare and promote patient health.

## 1. Introduction

Pressure injuries (PrIs) are areas of skin, and possibly tissue, that have been damaged by continuous pressure on specific body prominence areas. They are common adverse events in intensive care units (ICUs), and the majority of PrIs are preventable [1]. Patients who are admitted to ICUs with critical illnesses are often immobile and require bed rest; as a result, they are potentially prone to experiencing edema due to a poor venous return, circulation dysfunction, and hemodynamic instability with vasopressors. Some patients suffered from respiratory failure and required mechanical ventilation, agitation and required restraints, and exposure to moisture due to incontinence. The risk factors for PrIs are advanced age and obesity; the risk of PrIs increases as age and weight increase [2]. The incidence of adult PrIs ranges from 12% to 33% [3,4]. PrIs have significant impacts on patients, including pain, restricted mobility, wound infection, and psychological burden. They reduce quality of life and increase the risk of complications, resulting in longer hospital stays and increased health care costs [5].

The best way to prevent PrIs is to maintain clean and dry skin, avoid prolonged durations in the same position, and ensure the appropriateness of padding and mattresses to alleviate pressure. If PrIs are detected, prompt cleaning and management of the affected area should be undertaken in addition to necessary treatment. Due to the severity and complexity of their illnesses, critically ill patients are subjected to prolonged immobilization, have insufficient perfusion and poor nutritional statuses, and are at a higher risk for developing PrIs than general ward patients. Preventive interventions for PrIs focus on multiple factors and require the combination of various strategies to achieve optimal results. There was an association between the reduced risk of PrIs and preventive interventions, and bundle care is based on noteworthy, evidence-based interventions [6].

There are some care measures for and evidence on the prevention of PrIs. However, the implementation of prevention strategies is often not optimal [3,4]. Standard bundle care for PrI prevention can enable healthcare providers to control modifiable risk factors and enhance the quality of nursing care [7]. Previous studies have shown that the use of care bundles for skin care, including silicone dressing, skin protectants, comprehensive skin assessments from head to toe, heel offloading, the early identification of pressure sources, and repositioning, can effectively prevent PrIs by reducing the incidence density of PrIs [8,9,10]. The SSKIN (surface, skin investigation, kinetics/keep moving, incontinence/moisture, and nutrition/hydration) Care Bundle has been used frequently [11]. Previous studies found that the SSKIN bundle could effectively reduce the incidence of PrIs, improve patient outcomes, and enhance nurse compliance [12,13].

The main priority for nurses who administer critical care is the preservation of life quality during the treatment of life-threatening conditions rather than PrI prevention. Nurses with a heavy load take care of critical patients by monitoring vital signs and alerting physicians to life-threatening situations and have paid less attention to PrI prevention in the past. PrI prevention is an important issue of healthcare quality. Evidence-based critical care is necessary to prevent PrIs in the ICU. We used retrospective data from tertiary teaching hospitals in northern Taiwan before and after bundle care to evaluate the effect of PrI prevention to assess whether the trend of PrI incidence reduced.

The aim of this study was to evaluate the efficacy of care bundle use in PrI prevention by considering the influence on the incidence density of PrIs compared with the pre-bundle stage. Standardized and regular bundle care bundles for PrI prevention are designed by multidisciplinary teams to reduce PrI incidence and promote patient health. Such teams provide evidence on the benefits of care bundles for PrI prevention in the critical care setting.

## 2. Materials and Methods

### 2.1. Materials and Methods

This study was a review of the clinical data of adult ICU patients in Taipei Veterans General Hospital; there were 42 beds in the ICUs. The ICUs were the medical ICU and the surgical ICU. This study was a retrospective observation case–control study; the data were obtained from the nursing information system. The patients were hospitalized in ICUs from 1 January 2014 to 31 December 2019, in one medical center in northern Taiwan. The exclusion criteria were patients who underwent cardiac catheterization, and patients who were younger than 18 years of age. The patients that received cardiac catheterization on schedule had milder illnesses, and those younger than 18 years old had complex conditions. Data on sex, age, length of stay, body weight, comorbidities, disease severity, and use of sedatives, muscle relaxants, or inotropic agents were collected from the information system of ICUs.

The team who implemented bundle care for PrIs included critical care physicians, critical care nurses, wound care specialist nurses, and a member of the medical quality team who developed the SSKIN Care Bundle based on evidence in the literature [11]. Every patient who was admitted to ICUs was assessed by using the Braden Scale to determine their risk for PrIs [14], and high-risk patients were assigned to air suspension bed because the surface was supported with protection. The patients were repositioned every 2 hours and encouraged to move early, and the angle of their head did not exceed 30 degrees. To protect the urine or stool from irritating the skin, water was used for cleaning and the skin was kept dry. The nutrition evaluation was performed during hospitalization and twice every week to assess the patients’ intake of protein and calories. The items of the SSKIN care bundle are shown in Table 1. PrIs were defined by the European Pressure Ulcer Advisory Panel (EPUAP) and the National Pressure Ulcer Advisory Panel (NPUAP). A PrI is localized damage to the skin and/or underlying tissue, typically occurring over bony prominences, resulting from pressure with or without shear. Incidence density was calculated by dividing the number of new cases of PrIs by the total patient days of hospitalizations in the ICUs.

The care bundle for PrI prevention has been used in routine nursing care in critical care units since 1 January 2017. The care bundle for PrI care and prevention included good training and education before 2017. During the implementation phase, the nurse leader assessed the care bundle weekly to ensure compliance. Approximately 90% of nurses use the bundle. The primary outcome was the incidence density of PrIs. This study was approved by the Institutional Review Board (TPEVGH_IRB number: 2020-07-002CC on 24 June 2020).

### 2.2. Statistical Analysis

The distribution of categorical variables was analyzed using chi-square tests, while independent t tests were assessed for continuous data. Regarding exposure and nonexposure case numbers summed with an alpha of 0.05 and power of 80%, 1547 in each group and a total of 3094 was required. The periods were divided into two groups based on the time before (2014–2016) and after the implementation (2017–2019) of the target bundle care intervention. Poisson regression with log-linear regression for count data was performed to compare the incidence densities between two periods of all PrIs and different types of PrIs. *p* < 0.05 was considered to indicate a significant difference. The statistical analysis was performed using SPSS version 21.0 software.

## 3. Results

**Figure 1:**
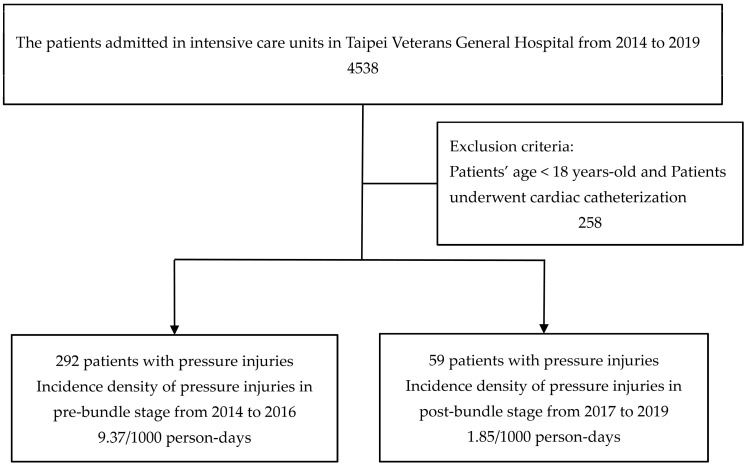
Flow chart of this study.

There were 4538 patients who were hospitalized in ICUs and data on a total of 64,171 patient day hospitalizations were obtained from the clinical information system. After excluding patients younger than age 18 and patients who received cardiac catheters (258 patients and 1031 patient day hospitalizations), the total number of patients was 4280 patients along with 63,140 patient day hospitalizations. The incidence density of PrIs was 9.37/1000 person days before the implementation of bundle care, and the incidence density of PrIs was 1.85/1000 person days after the implementation (*p* < 0.001) (Figure 1). The number of patients who required restraints in the ICU was significantly decreased. There were insignificant differences in sex, age, comorbidities, disease severity score, various laboratory values, and use of analgesics, sedatives, or vasopressors between the two stages (Table 2).

**Figure 2:**
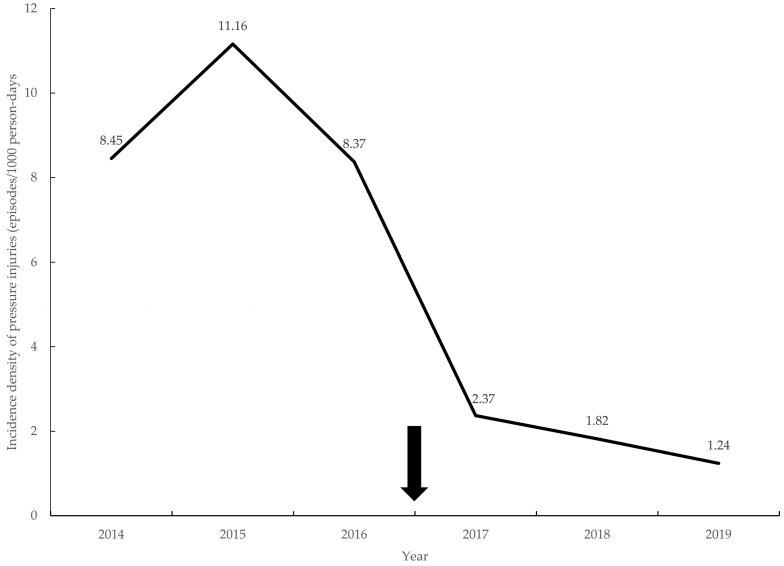
The incidence density of pressure injury in intensive care units. Bundle care implemented since 2017 (arrow).

Before the implementation of the care bundle, the highest incidence density of PrIs in the ICU was 11.16 episodes/1000 person days during 2015. However, the comprehensive implementation of the SSKIN care bundle began in 2017, and the incidence density significantly decreased to 2.37 episodes/1000 person days in 2017 and reached the lowest of 1.24 episodes/1000 person days in 2019. There was a significant reduction of 88.89% in the incidence density of PrIs between 2015 and 2019 (Figure 2).

**Figure 3:**
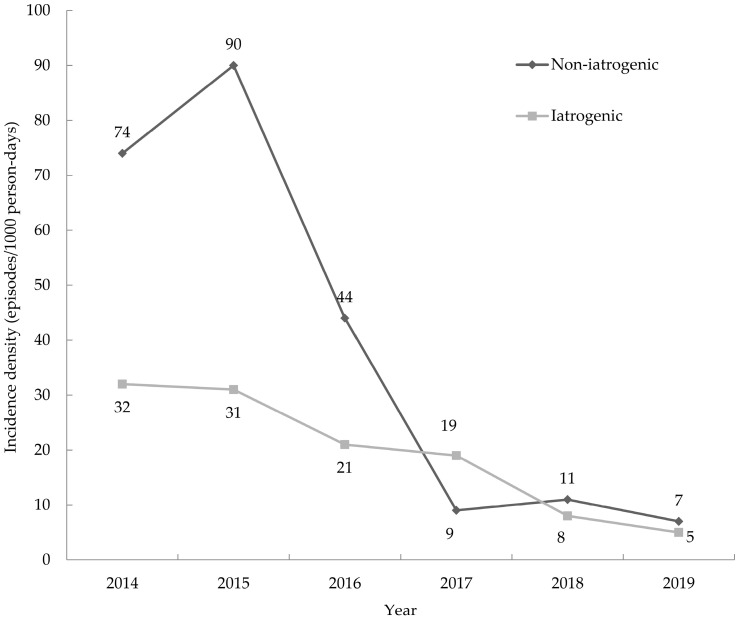
Cases of non-iatrogenic and iatrogenic pressure injuries in intensive care units.

The sacrum was the most common site of non-iatrogenic PrIs in the ICU before the implementation of the care bundle, followed by the ischial area and heels. The nose was the most common site of iatrogenic PrIs in the ICU, followed by the auricle in pre-bundle stage (Supplementary Table S1. The cases of different sites existing in iatrogenic and non-iatrogenic pressure injuries). The cases of iatrogenic and non-iatrogenic PrIs were significantly reduced after bundle care (Figure 3). The incidence density of non-iatrogenic PrIs significantly decreased from 6.67 to 0.84 episodes/1000 person days, with an RR of 0.126 (95% C.I.: 0.085–0.189, *p* < 0.001). The incidence density of iatrogenic PrIs significantly decreased from 2.7 to 1 episode/1000 person days, with an RR of 0.371 (95% C.I.: 0.247–0.558, *p* < 0.001) (Table 3).

The incidence density of PrIs in the ischium was the most significantly decreased, from 1.44 to 0.03 episodes/1000 person days with an RR of 0.022 (95% C.I.: 0.003–0.157, *p* < 0.001), and from 2.5 to 0.41 episodes/1000 person days with an RR of 0.162 (95% C.I.: 0.09–0.292, *p* < 0.001) in the sacrum as well as from 2.12 to 0.25 episodes/1000 person days with an RR of 0.118 (95% C.I.: 0.057–0.246, *p* = 0.001) in the lower limbs, but the reduction was not significant from 0.42 to 0.16 episodes/1000 person days with an RR of 0.375 (95% C.I.: 0.134–1.051, *p* = 0.062) in the back. The incidence density of PrIs in the nasal bridges was the most significantly decreased, from 0.55 to 0.06 episodes/1000 person days with an RR of 0.115 (95% C.I.: 0.026–0.496, *p* = 0.004); from 0.51 to 0.13 episodes/1000 person days with an RR of 0.244 (95% C.I.: 0.081–0.729, *p* = 0.012) in the face; and from 0.67 to 0.25 episodes/1000 person days with an RR of 0.371 (95% C.I.: 0.164–0.838, *p* = 0.017) in the auricle, but the reduction was not significant in the nasal wings, from 0.67 to 0.41 episodes/1000 person days with an RR of 0.603 (95% C.I.: 0.302–1.205, *p* = 0.152), and in the upper limbs, from 0.29 to 0.16 episodes/1000 person days with an RR of 0.541 (95% C.I.: 0.181–1.615, *p* = 0.271) (Table 3).

## 4. Discussion

This study found that the incidence of PrIs was significantly reduced after the implementation of care bundle intervention for 3 years. It was a new intervention with systemic prevention compared to previous routine nurse care in the Chinese population. In the long term, healthcare workers can fully understand the benefit of such measures that are designed to promote patient health by reducing the incidence of PrIs. Care bundles are essential to clinical practice. Although the overall iatrogenic and non- iatrogenic PrIs were decreased, there was still no significant reduction in the back, upper limbs, and nasal wings. They need more aggressive methods to reduce their incidence.

A recent study showed that integrating PrI prevention into practice significantly reduced the incidence of hospital-acquired PrIs (HAPI). The number of HAPI cases decreased from nine to one from the pre-intervention to post-intervention periods [15]. A prospective intervention using a prevention bundle was implemented in another study, and it significantly reduced the cumulative incidence rate of medical-device-related PrIs by 90% [16]. In our study, the number of PrI cases decreased from 121 to 12 (in 2015 vs. in 2019), resulting in a 90% reduction. A previous study found a significant reduction in the cumulative incidence density of PrIs in an intervention based on evidence-based skin care compared to the control group (*p* = 0.04, 18.1% vs. 30.4%) [17]. Another study observed a decrease in the incidence density of acquired PrIs from 15.5% before the intervention to 2.1% after the preventive intervention supported by wound ostomy continence nurses [9]. In our study, following the implementation of the care bundle, the incidence density of PrIs decreased from 9.37 to 1.85 episodes/1000 person days (*p* < 0.001), indicating that the care bundle for PrI prevention effectively reduces the occurrence of PrIs.

A multidisciplinary clinical and risk assessment team intervened for PrI prevention and used the SSKIN bundle care for PrI prevention. They found a reduction in the incidence of HAPI from 6.1‰ to 1.1‰, representing an 83.5% decrease [18]. Our study showed a similar finding, with a decrease of 80.26% of deceased patients. It emphasized that all patients should undergo a risk assessment within 2 h of admission and every 8 h thereafter. Furthermore, high-risk patients should be placed on pressure-relieving mattresses, undergo position changes at least every 2 h, and receive support to improve their nutritional status. For patients at a high risk, preventive dressings (sacrum, heels, hips) should be used to reduce the incidence of Prls in critically ill patients [19]. Our check time in every shift time was approximately 8 h, and there was a reduction in the incidence of PrIs when compared with the pre-intervention stage. Nutritional status should be evaluated after ICU admission by a nutritionist and twice every week during the ICU stay. The care bundle was established by a multidisciplinary team incorporating evidence-based knowledge and implemented in clinical practice for PrI prevention. The results enhanced the effectiveness of prevention efforts in overall PrIs and iatrogenic and non-iatrogenic PrIs.

Before the intervention, the most common sites of iatrogenic PrIs were the auricle and nasal wings, followed by the nasal bridge (Supplementary Table S1: The cases of different sites of non-iatrogenic and iatrogenic pressure injuries). After the implementation of the care bundle, the nasal wings were still the most common site with an insignificant reduction. Patients undergoing oral or gastrointestinal surgery or swallowing difficulties need nasal–gastric tubes, and endotracheal tubes from the nose or mouth inserted for respiratory failure must be properly fixed in the philtrum area, leading to persistent PrIs in the nasal wings. We added foam padding to the inner edge of the nasal wings beginning in 2018, and the number of PrIs in the nasal wings decreased from 2018 to 2019. We addressed PrIs caused by oxygen masks by increasing the length of foam padding and using more effective materials.

The main sites of PrIs were peripheral oxygen saturation monitors and splints (two cases). This potentially results in nursing staff, with a busy clinical workload, failing to adequately reposition finger-type oxygen saturation monitors after turning the patient. Ring-type oxygen saturation monitors replaced the finger-type monitors for patients with poor peripheral circulation after discussions with the monitor team and administrators and timely repositioning of the finger-type monitors. The close inspection of the skin condition at the bony prominence area was performed when removing the splint in a timely manner during each shift time, and foam padding was implemented to prevent PrIs caused by splints.

The most common sites of non-iatrogenic PrIs were the sacrum, followed by the ischium and heel before the care bundle intervention. After the care bundle intervention, PrIs in the ischium and heel were almost completely prevented. It emphasizes relieving pressure on vulnerable areas and bony prominences in any position and elevating the head of the bed while not exceeding 30 degrees. PrIs in the sacrum still existed with five sacral PrIs annually, which need more strategies to address. This potentially resulted from a higher acuity and severity of illness in ICU patients and some self-mobilized patients with an inadequate maintenance of the 30-degree positioning of pillows.

There was an insignificant reduction in incidence density in the back, nasal wings, and upper limbs. The potential reason was the maintenance of 26 °C in the public units to save electronic energy based on a government policy that caused the patients’ back to easily sweat when lying for a long duration. The colder temperature of air conditions could be set to reduce patients’ sweating. Because an air suspension bed is used for patients with a higher PrI risk, some patients with an intermittent risk may progressively develop PrIs. The wide usage of air suspension in ICUs could reduce the incidence of PrIs in the future. A nasogastric tube and endotracheal tube were fixed on nasal wings in the emergency room or general rooms before transfer to ICUs, and new technology needs to be promoted to other units of the hospital to further reduce the PrIs in nasal wings. In addition, the gastrectomy encouraged for long-term nasal–gastric feeding could reduce PrIs in nasal wings. The majority of intravenous injections and artery lines were performed in the upper limbs, while careful repositioning was needed to prevent compression and early change to a central-line insertion in the subclavian or femoral veins to reduce PrIs in the upper limbs. In addition, a smart clothing study to prevent PrIs is ongoing [20]. Although albumin insignificantly increased (2.89 to 3.1 g/dL), trend nutrition improved after frequent nutritionist support for patients in the ICUs.

The liability profile could be excluded by proper documentation of the adequacy of the precautionary measures [21]. Many interventions for PrIs may only be effective in the short term [6], and our study showed persistent effectiveness for 3 years. The care bundle intervention proposed in this study is an effective approach for preventing PrIs in critically ill patients. Furthermore, this study brought about significant policy changes by only shifting from the traditional practice of repositioning every 2 hours (left side, right side, supine) to a concept of integrated intervention avoiding pressure on specific sites. At the initial implementation of the care bundle in the clinical practice of critical care, there was some resistance due to the increased workload by changing existing beliefs and habits with significant pushback. Through continuous communication with interdisciplinary collaboration to focus on the directions of enhancing patients’ safety and improving the quality of healthcare, colleagues gradually became more accepting of changing their beliefs. The knowledge and skills regarding the care bundle were consistently taught during on-the-job training and prevocational education. The psychometric properties of the Pressure Ulcer Management Self-Efficacy Scale of nurses related to the care of PrIs in Taiwan [22] were included. A balanced distribution of nurses would improve the quality of care for PrIs [23]. Bundle care could be successful based on a nursing team designed with consulting expertise, reducing the barriers to promoting standard forms of care into regular care with good education and feedback audits to establish nursing facilities for the care of PrIs [13].

There are some limitations in our study. First, this study utilized a retrospective review of electronic health records. There were some missing or incomplete data due to various reasons, and the incidence density of PrIs may be underestimated. Second, there was a trend change in this study rather than a direct causal relationship between the care bundle and the reduction in the incidence density of PrIs. Third, the severity of PrIs was not surveyed. Fourth, the IRB granted approval for a retrospective study before 2020, and the incidence density persistently decreased by 1.2, 0.4, and 1.09/1000 person days from 2020 to 2022, respectively. Fifth, the confounding factors were not adjusted in Poisson regression, and only a mild reduction in restraint requirement after bundle care was performed. Further logistic regression models need to include confounding factors.

## 5. Conclusions

This study found increasing evidence that integrated care by translating evidence-based knowledge into clinical practice can effectively reduce PrI incidence to improve the quality of care and patients’ health outcomes. The PrI care bundle for critical care patients was redesigned through multidisciplinary teamwork and organized structures with effective communication. Therefore, through integrated care through education and training, nurses can take steps to provide reliable clinical care and reduce the occurrence of PrIs.

## Figures and Tables

**Table 1:** The items and actions of SSKIN bundle care of pressure injuries’ prevention.

Items	Action
Surface	Air suspension bed for patients with higher risk of pressure injuries. The others use a pressure-reduced bed. When lying flat, place a pillow under both knees and calves to keep the heels off the bed. When lying on the side, place a pillow (at a 30-degree angle) behind the back to relieve pressure on the coccyx. The shoulder and hip joints on the side should be slightly tilted outward to relieve pressure. Bend the leg on the upper side, place a pillow between the knees, and ensure that the knees are not under pressure and the ankles are elevated. After turning over, adjust the position of the head and place a rolled towel behind the ear. Artificial skin pressure reduction products should be used at the pressure points when using a nasal intermittent positive pressure ventilation and loosened every 2 hours to inspect the skin. Ensure nasogastric tube or endotracheal tube fixed with Ω sharp without stressing nasal wings, and oxygen mask without pressuring nasal bridges or auricles with foam dressings.
Skin investigation	New patients and during reposition period, a real-time “head-to-toe” assessment of overall skin temperature, color, moisture status, and integrity, with particular attention to bony prominences. During each shift period, the overall risk for pressure injuries was assessed using the Braden Scale [1], which includes factors such as sensory perception, moisture, activity, mobility, nutrition, and friction/shear. A score of ≤14 is used to identify patients at high risk for pressure injuries. Check that the tubes are not under pressure by skin. Avoid compressing area that includes redness and refrain from massaging bony prominences.
Kinetics/keep moving	The turning schedule and prohibited actions were strictly followed to assist in changing the patient’s position and limb placement correctly every 2 h. Encourage early movement, perform physical therapy, relieve spasticity, and limit sedative use. The bed should be leveled before turning the patient, and grasp the turning sheet closer to the patient’s side and lift, avoiding pushing or pulling. After turning over, raise the foot end of the bed before elevating the head end, ensuring that the angle does not exceed 30 degrees and replace the position of the pulse oximeter.
Incontinence/moisture	Identify the stage of incontinence-associated dermatitis and fungal infection. Cleanse the skin affected by incontinence with water. To protect irritable skin from the urine or stool, keep skin clean and dry.
Nutrition/hydration	The nutritionists evaluated each patient after hospitalization and assessed their nutritional statuses twice every week to ensure adequate intake of protein and calories.

**Table 2:** Comparison of control variables between the two periods.

Variables	Pre-Bundle Stage (n = 2134)	Post-Bundle Stage (n = 2146)	*p*
The cases of PrIs	292	59	<0.001 *
Incidence density (/1000 person days) of PrIs	9.37	1.85	<0.001 *
Sex (Male)	1356 (63.54%)	1345 (62.67%)	0.539
Age	67.18 ± 17.53	67.55 ± 15.98	0.839
Length of stay	14.60 ± 11.52	14.90 ± 12.55	0.555
Glasgow Coma scale	10.84 ± 4.111	10.88 ± 3.927	0.805
Body weight	62.86 ± 14.57	63.48 ± 14.83	0.179
Albumin (g/dL)	2.886 ± 0.597	3.100 ± 0.592	0.214
Ventilation	8.422 ± 7.466	8.205 ± 7.800	0.419
Potassium (mEq/L)	3.977 ± 0.813	3.921 ± 0.718	0.407
Sodium (mEq/L)	139.8 ± 7.491	140.3 ± 7.272	0.694
Calorie achievement rate	72.57 ± 21.19	69.37 ± 22.48	0.722
Charlson comorbidity index	5.29 ± 2.32	5.02 ± 2.27	0.184
APACHE II score within first day	21.20 ± 7.03	22.32 ± 8.36	0.169
APACHE II score	20.21 ± 7.58	19.75 ± 8.45	0.175
Pain scores	1.64 ± 1.52	1.49 ± 1.96	0.360
Incontinence-associated dermatitis	447 (20.95%)	462 (21.53%)	0.642
Restraints	1908 (89.41%)	1871 (87.19%)	0.024 *
Sedation	1410 (66.07%)	1473 (68.64%)	0.073
Muscle relaxant	32 (1.5%)	31 (1.44%)	0.881
Inotropic agents	368 (17.24%)	372 (17.33%)	0.938
Pain control	1651 (77.37%)	1669 (77.78%)	0.75
Ventilation	1944 (91.1%)	1931 (89.98%)	0.213
Life support system	115 (5.39%)	112 (5.22%)	0.804
Nurse–patient ratio	2.53 (0.01)	2.6 (0.02)	0.374

* *p* < 0.05; PrIs: pressure injuries; APACHE II score: Acute Physiology and Chronic Health Evaluation II score. Life support system included Extracorporeal Membrane Oxygenation, Intra-Aortic Balloon Pump, and Continuous Venous Hemofiltration.

**Table 3:** The incidence density and relative risk of different types of pressure injuries before and after care bundle implementation.

	Pre-Bundle Stage Incidence Density (/00)	Post-Bundle Stage Incidence Density (/00)	Relative Risk	*p*
Overall PrIs	9.37	1.85	0.197 (95% C.I.: 0.149–0.26)	<0.001 *
Non-iatrogenic PrIs	6.67	0.84	0.126 (95% C.I.: 0.085–0.189)	<0.001 *
Sacrum	2.5	0.41	0.162 (95% C.I.: 0.09–0.292)	<0.001 *
Back	0.42	0.16	0.375 (95% C.I.: 0.134–1.051)	0.062
Ischium	1.44	0.03	0.022 (95% C.I.: 0.003–0.157)	<0.001 *
Occiput	0.19	0		
Low limbs	2.12	0.25	0.118 (95% C.I.: 0.057–0.246)	0.001 *
Knee	0.35	0.03	0.089 (95% C.I.: 0.011–0.686)	0.02 *
Ankle	0.45	0.13	0.278 (95% C.I.: 0.092–0.846)	0.024 *
Heel	1.32	0.09	0.071 (95% C.I.: 0.022–0.23)	<0.001 *
Iatrogenic PrIs	2.7	1	0.371 (95% C.I.: 0.247–0.558)	<0.001 *
Nose	1.22	0.47	0.385 (95% C.I.: 0.212–0.699)	0.002 *
Nasal wings	0.67	0.41	0.603 (95% C.I.: 0.302–1.205)	0.152
Nasal bridge	0.55	0.06	0.115 (95% C.I.: 0.026–0.496)	0.004 *
Auricle	0.67	0.25	0.371 (95% C.I.: 0.164–0.838)	0.017 *
Face	0.51	0.13	0.244 (95% C.I.: 0.081–0.729)	0.012 *
Upper limbs	0.29	0.16	0.541 (95% C.I.: 0.181–1.615)	0.271

* *p* < 0.05. PrIs: pressure injuries; 95% C.I.: 95% confidence interval.

## Data Availability

The datasets used in the current study are available from the corresponding author upon reasonable request.

## Supplementary Materials

The following supporting information can be downloaded at: https://www.mdpi.com/article/10.3390/healthcare11152116/s1, Table S1: The cases of different sites of non-iatrogenic and iatrogenic pressure injuries.
