# Supplementary material for: Reduction in the Incidence Density of Pressure Injuries in Intensive Care Units after Advance Preventive Protocols
Source: Healthcare (Basel). 2023 Jul 25;11(15):2116. doi: 10.3390/healthcare11152116 (PMC10418660; doi:10.3390/healthcare11152116)
Supplement: Supplementary file 1 [file healthcare-11-02116-s001.zip › healthcare-2485001-supplementary.pdf]

Table S1. The cases of different sits of non-iatrogenic and iatrogenic pressure injuries.

Non-iatrogenic PrIs	2014	2015	2016	Pre-bundle stage	2017	2018	2019	Post-bundle stage	*p*
Sacrum	17	38	23	78	5	5	3	13	<0.001
Ischium	32	11	2	45	0	1	0	1	
back	5	5	3	13	1	2	2	5	
Knee/Hip/Shoulder	4	4	3	11	0	0	1	1	
Ankle	5	7	2	14	2	1	1	4	
Heel	7	23	11	41	1	2	0	3	
Occiput	4	2	0	6	0	0	0	0	
Total non-iatrogenic Pis	74	90	44	208	9	11	7	27	
Iatrogenic PrIs	2014	2015	2016		2017	2018	2019		*p*
Auricle	10	8	3	21	3	5	0	8	0.001
Nasal wings	4	4	13	21	10	2	1	13	
Nasal bridge	7	7	3	17	1	1	0	2	
Face	7	8	1	16	3	0	1	4	
Finger/Elbow/Arm	4	4	1	9	2	0	3	5	
Total iatrogenic PIs	32	31	21	84	19	8	5	32	

PrIs: pressure injuries.
